# Comprehensive analysis of lung macrophages and dendritic cells in two murine models of allergic airway inflammation reveals model- and subset-specific accumulation and phenotypic alterations

**DOI:** 10.3389/fimmu.2024.1374670

**Published:** 2024-03-11

**Authors:** Belinda Camp, Ilka Jorde, Franka Sittel, Alexander Pausder, Andreas Jeron, Dunja Bruder, Jens Schreiber, Sabine Stegemann-Koniszewski

**Affiliations:** ^1^ Experimental Pneumology, Department of Pneumology, University Hospital Magdeburg, Health Campus Immunology, Infectiology and Inflammation, Otto-von-Guericke University, Magdeburg, Germany; ^2^ Infection Immunology Group, Institute of Medical Microbiology, Infection Control and Prevention, University Hospital Magdeburg, Health Campus Immunology, Infectiology and Inflammation, Otto-von-Guericke University, Magdeburg, Germany; ^3^ Department of Pediatrics, Ludwig-Maximilians University of Munich, Munich, Germany; ^4^ Immune Regulation Group, Helmholtz Centre for Infection Research, Braunschweig, Germany

**Keywords:** allergic asthma, allergic airway inflammation, macrophages, dendritic cells, spectral flow cytometry, murine model

## Abstract

**Introduction:**

Allergic asthma has been mainly attributed to T helper type 2 (Th2) and proinflammatory responses but many cellular processes remain elusive. There is increasing evidence for distinct roles for macrophage and dendritic cell (DC) subsets in allergic airway inflammation (AAI). At the same time, there are various mouse models for allergic asthma that have been of utmost importance in identifying key inflammatory pathways in AAI but that differ in the allergen and/or route of sensitization. It is unclear whether and how the accumulation and activation of specialized macrophage and DC subsets depend on the experimental model chosen for analyses.

**Methods:**

In our study, we employed high-parameter spectral flow cytometry to comprehensively assess the accumulation and phenotypic alterations of different macrophage- and DC-subsets in the lung in an OVA- and an HDM-mediated mouse model of AAI.

**Results:**

We observed subset-specific as well as model-specific characteristics with respect to cell numbers and functional marker expression. Generally, alveolar as opposed to interstitial macrophages showed increased MHCII surface expression in AAI. Between the models, we observed significantly increased numbers of alveolar macrophages, CD103^+^ DC and CD11b^+^ DC in HDM-mediated AAI, concurrent with significantly increased airway interleukin-4 but decreased total serum IgE levels. Further, increased expression of CD80 and CD86 on DC was exclusively detected in HDM-mediated AAI.

**Discussion:**

Our study demonstrates a model-specific involvement of macrophage and DC subsets in AAI. It further highlights spectral flow cytometry as a valuable tool for their comprehensive analysis under inflammatory conditions in the lung.

## 1 Introduction

Asthma is one of the most common chronic respiratory diseases and affects more than 300 million patients worldwide (1, 2). Generally, asthma is characterized by airway inflammation, bronchial hyperreactivity, mucus overproduction, variable airway narrowing and airway wall remodeling (3). Different phenotypes and endotypes have been defined for this extremely heterogeneous disease, also with respect to the associated chronic inflammation. A major discrimination is that between non-allergic (intrinsic) and allergic (extrinsic) asthma. Most children and around 50% of adult asthmatics suffer from allergic asthma (1). Here, initial exposure to an allergen is typically associated with T helper type 2 (Th2) responses. These include the secretion of cytokines such as interleukin-4 (IL-4), IL-5 and IL-13 (1). Elevated IgE titers and eosinophilia characterize the disease (4, 5) and also neutrophils can be involved (1). To improve prevention and therapy, there is a large interest in defining predisposing factors as well as factors relevant to the manifestation of different inflammatory phenotypes together with their immunological way of action. Next to the central role of T lymphocytes in allergic type 2 immune responses, there is an increasing awareness for contributions of myeloid cells such as dendritic cells (DC) and macrophages in regulating inflammatory processes in allergic asthma.

In mice and humans, tissue resident macrophages are myeloid sentinel cells located in all organs. Resident macrophages in mucosal tissues have received increasing attention and numerous studies have demonstrated that these cells play critical roles in maintaining and restoring tissue immune homeostasis (6, 7). It is well established that the lung harbors two distinct populations of macrophages: alveolar macrophages (AMs) and interstitial macrophages (IMs), with IMs dividing in several subsets (8). In contrast to AM located in the alveoli, IMs are typically located in the interstitium along with DC and lymphocytes. IMs have been classified into several distinct populations, mainly based on their phenotype (8). However, macrophage classification is complex and displays a dynamic field varying between reports. Likewise, knowledge of the functions of different IM subpopulations during homeostasis, infection or inflammatory conditions is still limited. A distinct subset of IMs are CD169^+^ nerve- and airway associated macrophages (NAMs) that are morphologically and transcriptionally distinct from AMs. NAMs are lung-resident macrophages harboring regulatory functions and are primarily localized around the large bronchiolar airways adjacent to airway-associated nerves (9). Their role in allergic asthma is unknown to date. Next to NAMs, IMs can be divided into CD11c^+^ and CD11c^-^ IMs, which are both negative for CD169, separating them from NAMs. Major histocompatibility class (MHC)II complex is required for antigen-presentation, critical in the regulation of immune responses and expressed on the surface of macrophages (10).

In addition to macrophages, DC also play an important role in inducing Th2 immunity toward inhaled antigens (11). Literature has demonstrated that DC can be divided in several subgroups with specific functions (12). In the murine lung, conventional DC (cDC) can be subdivided into CD103^+^ and CD11b^+^ cells (13). Further, monocytes can differentiate to monocyte-derived DC under inflammatory conditions, such as in asthma (14). In mice, cDC are essential for the migration and induction of differentiation of Th2 cells in the lung draining lymph nodes upon allergen exposure (14). However, there is substantial controversy as to the involvement of specific cDC subsets in atopic asthma in mice and humans. In the light of their efficiency in antigen processing and presenting together with their ability to induce Th2 responses, CD11b^+^ cDC2 are thought to be key to the induction of allergic airway inflammation. In contrast, the role of CD103^+^ cDC1 is less clear and there are conflicting reports as to whether CD103^+^ DCs accumulate in the lung in AAI and promote or alleviate inflammation (15).

Mouse models are of central importance in elucidating the roles of innate and adaptive immune cells, key cytokines, cellular and soluble mediator networks and pathways in both the development and the effector phase of allergic asthma. Several models are available, differing in the choice of the allergen, the need for adjuvants and the route of sensitization. Murine models using ovalbumin (OVA) as a model antigen have long been mainstay in analyzing Th2 immunity. To experimentally induce allergic airway inflammation (AAI), mice are first immunized systemically with OVA through intraperitoneal (i.p.) injection, typically together with the adjuvant aluminium hydroxide (alum), to induce a Th2-driven OVA-specific immune response. Sensitization is followed by OVA-challenges administered over the airways, resulting in a specific IgE response, acute airway inflammation and establishment of airway hyperreactivity (AHR) (16). However, OVA-mediated AAI often harbors differences in the underlying pathophysiology between mice and human patients (17). Therefore, additional models of acute allergic asthma have been validated using naturally occurring allergens, such as house dust mite (HDM). HDM-mediated AAI relies on respiratory sensitization without the need for an adjuvant and mimics human disease more closely than OVA-mediated AAI (18). Model-specific manifestations of inflammation can lead to different results and outcomes of intervention. In this regard, fairly little is known with respect to model-specific characteristics of the subset-specific involvement of myeloid cells in experimentally induced AAI.

Flow cytometry has emerged as a critical tool for studying immune cell populations in the lung and other organs. Especially in the lung, cellular autofluorescence (AF) of macrophages and eosinophil granulocytes can interfere with the signals of fluorescent markers, resulting in poor resolution or false positive results (19, 20). Spectral flow cytometry acquires complete spectral emission signatures and subsequent analysis allows the identification, characterization and handling of lung AF signals within a complex mixture of cell types. Using high-parameter spectral flow cytometry, we comprehensively assessed the involvement of different subsets of lung macrophages and DC in an OVA- and an HDM-mediated model of experimentally induced AAI. This systematic analysis revealed distinct differences in the accumulation of myeloid cell subsets and their activation between the models. These differences should play a decisive role for model selection and need to be considered in the interpretation of data obtained from a certain AAI model.

## 2 Material and methods

### 2.1 Mice

All experiments were performed in 7-8 week-old female specific-pathogen free C57Bl/6JRj mice. Mice were obtained from Janvier (Saint-Berthevin, France), housed in individually ventilated cages in groups of 3-5 and were fed food and water ad libitum. All experiments were ethically reviewed and approved by the responsible authorities (Landesverwaltungsamt Sachsen-Anhalt, file number 203.6.3-42502-2-1495).

### 2.2 Induction of allergic airway inflammation

#### 2.2.1 Ovalbumin-mediated AAI

**Figure 1:**
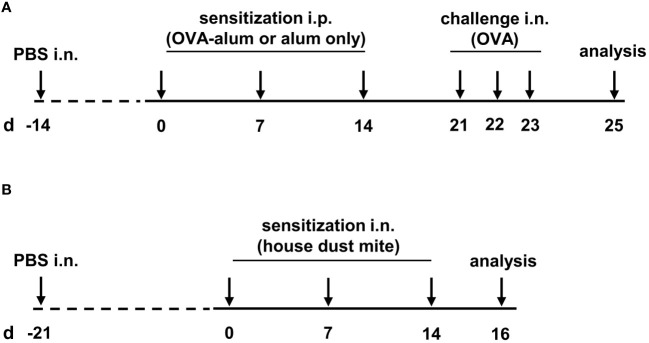
Timeline of the experimental setups. **(A)** For the induction of ovalbumin-mediated allergic airway inflammation (OVA-AAI), mice were sensitized intraperitoneally (i.p.) with 10 µg ovalbumin (OVA) and aluminium hydroxide (alum) three times in weekly intervals (d 0, 7, 14). One week after the last sensitization they were intranasally (i.n.) challenged with OVA alone (100 µg) on three consecutive days (d 21, 22, 23). **(B)** For house dust mite mediated allergic airway inflammation (HDM-AAI), mice were treated i.n. with 100 µg house dust mite (HDM) three times in weekly intervals (d 0, 7, 14).

As a measure of the reduction of experimental animal use, the animals reported here served as control groups in other experiments and were intranasally (i.n.) treated with 25 µl PBS once before the first OVA sensitization (day -14). From two weeks after control treatment with PBS, mice were sensitized i.p. with 10 µg ovalbumin (OVA, grade V, Sigma-Aldrich, St. Louis, MO, USA) in PBS containing 1 mg aluminium hydroxide (alum; Imject™ Alum Adjuvant, Thermo Fisher) in three weekly intervals (day 0, 7 and 14). Control mice were mock-sensitized i.p. with alum only on days 0, 7 and 14. One week after the last sensitization, on three consecutive days (day 21, 22 and 23) all mice were intranasally (i.n.) challenged with 100 µg OVA (grade III, Sigma-Aldrich) in 30 µL PBS under light isoflurane anesthesia. Forty-eight hours after the last challenge (day 25), all mice were sacrificed and bronchoalveolar lavage (BAL), serum and lungs were harvested for further analyses (for timeline see **Figure 1A**).

#### 2.2.2 House dust mite-mediated mouse model

Three weeks after control treatment with PBS (day -21; see above), mice were treated i.n. with 100 µg house dust mite (HDM, Stallergenes Greer, Lenoir, USA) in 50 µl PBS three times in weekly intervals (day 0, 7, 14). Control mice were treated i.n. with 50 µl PBS. Forty-eight hours after the last treatment (day 16), mice were sacrificed and BAL, serum and lungs were harvested for further analysis (for timeline see **Figure 1B**).

### 2.3 Serum

Blood was collected post mortem, incubated for 20 min at 37°C and 5 min at 4°C and centrifuged for 10 min at 4°C (1500 x g). Serum was aliquoted and stored at -80°C until further analysis.

### 2.4 Recovery of BAL and isolation of lung leukocytes

BAL was obtained by flushing the lungs once with 1 mL ice-cold PBS through the trachea. Subsequently, lungs were perfused with 10 mL ice-cold PBS through the heart to remove blood from the tissue, excised and minced on ice. Tissue degradation was performed by enzymatic digestion (45 min at 37°C) in Iscove´s modified Dulbecco´s medium containing 0.2 mg/mL Collagenase D (Sigma-Aldrich), 0.01 mg/mL DNase (Sigma-Aldrich) and 5% fetal calf serum. EDTA was added to a final concentration of 5 mM and suspensions were filtered (100 µm). Erythrocyte lysis by osmotic shock was performed and leukocytes were enriched using Percoll/NaCl (1.041 g/mL) (GE Healthcare Life Sciences).

### 2.5 Spectral flow cytometry

Lung leukocytes were incubated with anti-CD16/CD36 (2.4G2) to block Fc-receptors and simultaneously stained with fixable live/dead stain (BioLegend). Antibody staining for CD64 (X54-5/7.1) BV421, CD8a (53-6.7) BV510, CX3CR1 (SA011F11) BV605, CD80 (16-10A1) BV650, MHCII (M5/114.15.2) BV711, Ly6C (HK1.4) BV785, CD4 (GK1.5) Kiravia Blue 520, CD45 (30-F11) Spark Blue 574, SiglecF (S17007L) perCP/Cy5.5, CD86 (A17199A) PE, CD11b (M1/70) Spark yellow/green 593, CD169 (3D6.112) PE/Dazzle 594, F4/80 (QA17A29) PE/Fire 640, CD24 (30-F1) PE/Cy7, CD11c (QA18A72) PE/Fire810, MerTK (2B10C42) APC, CD103 (2E7) AF647 and Ly6G (1A8) AF700 was performed. Antibodies were obtained from BioLegend. Data were acquired using the Sony spectral analyzer ID7000 (Sony) equipped with 4 lasers (405 nm, 488 nm, 561 nm and 637 nm). The spectral unmixing and substraction of autofluorescence signals were performed using the Sony ID7000™ software, then unmixed FCS files were exported and analyzed using the FlowJo software (Tree Star) (see **Supplementary Material**-**Supplementary Figure 1**). Single stainings were performed for all fluorochromes for compensation using UltraComp eBeads (Thermo Fisher). For the calculation of absolute cell numbers from the relative frequencies, 50,000 fluorescent beads (Precision Count Beads, BioLegend) were added to each sample. Following singlet-gating and dead cell exclusion, cell populations were gated as follows: Live single CD45^+^ cells were gated for MerTK^+^/CD64^+^ cells. MerTK^+^/CD64^+^ cells were further gated for Ly6C^-^ cells and these then divided into AMs (CD11c^+^/CD169^+^) and remaining cells. From the remaining cells, CD11c^+^ IMs (CD11c^+^/CD169^-^/CX3CR1^+^), CD11c^-^ IMs (CD11c^low^/CD169^-^/CX3CR1^+^) and CD11c^low/+^ NAMs (CD11c^-/low^/CD169^high^/CX3CR1^+^) were gated. Remaining cells from the CD64/MerTK gating were further gated into CD103^+^ DC (CD11c^+^/CD11b^-^/CD103^+^/CD24^+^), neutrophils (CD11b^+^/Ly6G^+^) and eosinophils (CD11b^+^/CD11c^-^/Ly6G^-^/SiglecF^+^). Remaining cells from the SiglecF/CD11c gating that were not eosinophils were gated for CD11b^+/high^ cells. From these, MHCII^+^ cells were gated. From the CD11b^+/high^/MHCII^+^ cells, monocyte-derived recruited macrophages (CD11b^+/high^/MHCII^+^/CD64^+^) and CD11b^+^ DC (CD11b^+/high^/MHCII^+^/CD11c^+^/CD64^-^) were gated. For monocytes, CD11b^+/high^ cells were gated for MHCII^-^CD64^-^ cells that were further divided into Ly6C^+^ monocytes and Ly6C^-/low^ cells. Ly6C^-/low^ cells were gated for CXCR31^+^/Ly6C^-^ monocytes. T cells were gated as CD11b^-^/CD11c^-^/CD24^-^/MHCII^-^ and B cells were gated as CD11b^-^/CD11c^-^/MHCII^+^/CD24^low/+^ (see **Supplementary Material**, **Supplementary Figure 2**, **Supplementary Table 1**).

Manually gated populations from FACS data were exported into a combined master fcs file with a population label for each event. 27384 events per sample were contained in the master fcs file. Data in each fluorescence channel were transformed with the Yeo–Johnson algorithm (21) followed by an heuristic negative-peak detection algorithm. Z-scores in reference to the peak center of each channel’s negative peak were calculated in order to normalize fluorescence channels. Dimension reduction was performed using UMAP algorithm (22) (n_neighbors = 30 and min_dist = 0.2). Population assignment of manually gated events was color-coded. All calculations were performed using a Python script which is available upon reasonable request.

### 2.6 Enzyme-linked immunosorbent assay

Total serum immunoglobulin E (IgE) and serum albumin in BAL were quantified by ELISA. A 96-well ELISA plate (Fisher Scientific) was coated with the respective capture antibody (rat anti-mouse IgE, clone 23G3, SouthernBiotech; goat anti-mouse albumin, Biomol) and incubated over night at 4°C. To prevent non-specific binding, wells were blocked with assay diluent for 1 h at room temperature (RT) on a plate shaker. After incubation and washing steps, samples were added in duplicate (for IgE ELISA in 1:10 dilution, for albumin ELISA in 1:2000 dilution for control group samples and in 1:10000 for AAI samples) (negative control: assay diluent), incubated for 1 h (albumin) or 2 h (IgE) at RT on a plate shaker. The respective HRP conjugated detection antibody (IgE: goat anti-mouse, Biomol; albumin: goat anti-mouse, Thermofisher) was added. The tetramethylbenzidine (TMB) substrate set was mixed according to the manufacturer´s instructions (BD Bioscience). Substrate was added to all wells and incubated in the dark. The enzymatic reaction was stopped by adding furic acid (2N). The optical density (OD) was measured at 450 nm (albumin) or at 450 and 570 nm (IgE) (Tecan Infinite^®^ M Plex Photometer). Data were analyzed using Microsoft Excel and Graph Pad Prism software version 9 (Graph Pad Software). For analysis of serum albumin, a standard curve was generated using the absorbance of the standard wells and their known concentration (albumin from mouse serum, Merck KGaA). The concentration (ng/ml) of serum albumin in sample wells was determined from the acquired absorbance and the standard curve. For the analysis of total IgE the OD values were used to calculate the relative levels of the target substance.

### 2.7 Quantification of cytokines in BAL

Cytokines were quantified in undiluted BAL supernatant in duplicates using a 12-plex cytometric bead array according to the manufacturer´s instructions (LEGENDplex™ Th cytokine panel, BioLegend). IFN-γ (0.56 pg/mL), IL-5 (1.72 pg/mL), TNF-α (2.63 pg/mL), IL-2 (1.03 pg/mL), IL-6 (2.46 pg/mL), IL-4 (0.71 pg/mL), IL-10 (10.523 pg/mL), IL-9 (1.60 pg/mL), IL-17A (0.70 pg/mL), IL-17F (1.47 pg/mL), IL-22 (3.57 pg/mL) and IL-13 (1.05 pg/mL) were analyzed (detection limits). Values that were below the detection limit were evaluated as zero.

### 2.8 Statistical analysis

Data for all experimental groups were tested for normality using the Shapiro-Wilk normality test. In the case of Gaussian distribution for all groups in a comparison, an unpaired two-sided t-test was performed. In the case of non-Gaussian distribution in at least one of the groups in a comparison, an unpaired two-sided Mann-Whitney test was performed. p ≤ 0.05 was considered indicative for statistical significance (*p < 0.05, **p < 0.01, ***p < 0.005, ****p < 0.0001). All statistical analyses were performed using the Graph Pad Prism software version 9 (Graph Pad Software).

## 3 Results

### 3.1 OVA-AAI and HDM-AAI lead to similar eosinophil and neutrophil recruitment to the respiratory tract but to model-specific intensities of IgE- and respiratory cytokine-production

To compare AAI induced via peripheral sensitization towards a model antigen with that induced via respiratory sensitization towards a natural antigen, an OVA-AAI and an HDM-AAI mouse model were employed (**Figures 1A, B**). As a basis for the analysis of macrophage and DC subsets in these models, we performed a characterization of key inflammatory parameters in AAI, i.e. overall cell numbers, eosinophils and neutrophils in the lung, the IgE response, respiratory cytokine responses and respiratory vascular leakage.

**Figure 2:**
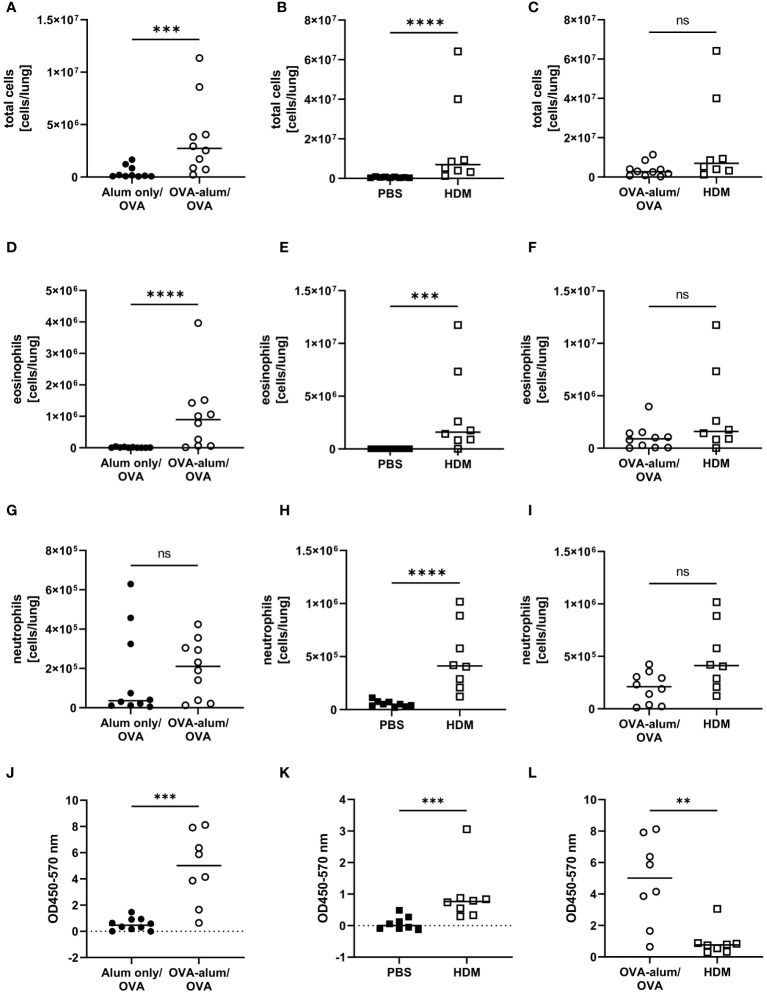
Accumulation of leukocytes, eosinophils and neutrophils in the lung and serum IgE production in OVA-AAI and HDM-AAI. For the induction of allergic airway inflammation (AAI), mice were treated with ovalbumin (OVA; n=10) (and aluminum hydroxide (alum) for sensitization) or house dust mite extract (HDM; n=8) as described in materials and methods. Control mice were mock-sensitized with alum only (OVA-AAI; n=10) or treated with PBS only (HDM-AAI; n=9). Lung leukocytes were analyzed by spectral flow cytometry and compared between OVA-AAI and HDM-AAI regarding total cell counts **(A-C)**, eosinophil numbers **(D-F)** and neutrophil numbers **(G-I)**. Serum IgE levels **(J, K)** were assessed by ELISA and compared between models **(L)**. Data compiled from at least three independent experiments are shown for individual mice with the median. **p < 0.01, ***p < 0.005, ****p < 0.0001, ns, not significant.

Due to different timelines and different treatments of the control animals between the models, the induction of OVA-AAI and HDM-AAI was separately evaluated in relation to the corresponding control group. Subsequently, the OVA-AAI and HDM-AAI groups were directly compared with each other in separate analyses. Both, in OVA-AAI and HDM-AAI, we observed a significant increase in total cell numbers in the lung as compared to control-treated mice (**Figures 2A, B**). Moreover, there was a trend for higher lung cell numbers in HDM-AAI as compared to OVA-AAI, which however did not reach statistical significance (p = 0.0676; **Figure 2C**). Eosinophils are key players in allergic asthma and also neutrophilic inflammation occurs to a varying extent. The numbers of lung eosinophils were significantly elevated in both OVA-AAI and HDM-AAI as compared to the respective control group (**Figures 2D, E**) and the increase in eosinophils did not differ significantly between both models (**Figure 2F**). At the same time there was a significant increase of neutrophils in HDM- but not OVA-AAI (**Figures 2G, H**), resulting in a trend for higher neutrophil numbers in HDM-AAI but no statistically significant difference between both models (p = 0.0545; **Figure 2I**).

Elevated levels of serum IgE display a hallmark characteristic of allergic asthma that likewise occurs in mouse models. Indeed, total serum IgE was significantly elevated in OVA-AAI as well HDM-AAI (**Figures 2J, K**). A comparison between median serum IgE levels in both models however revealed, that serum IgE levels were significantly higher in OVA-AAI as compared to HDM-AAI (5-fold; **Figure 2L**).

**Figure 3:**
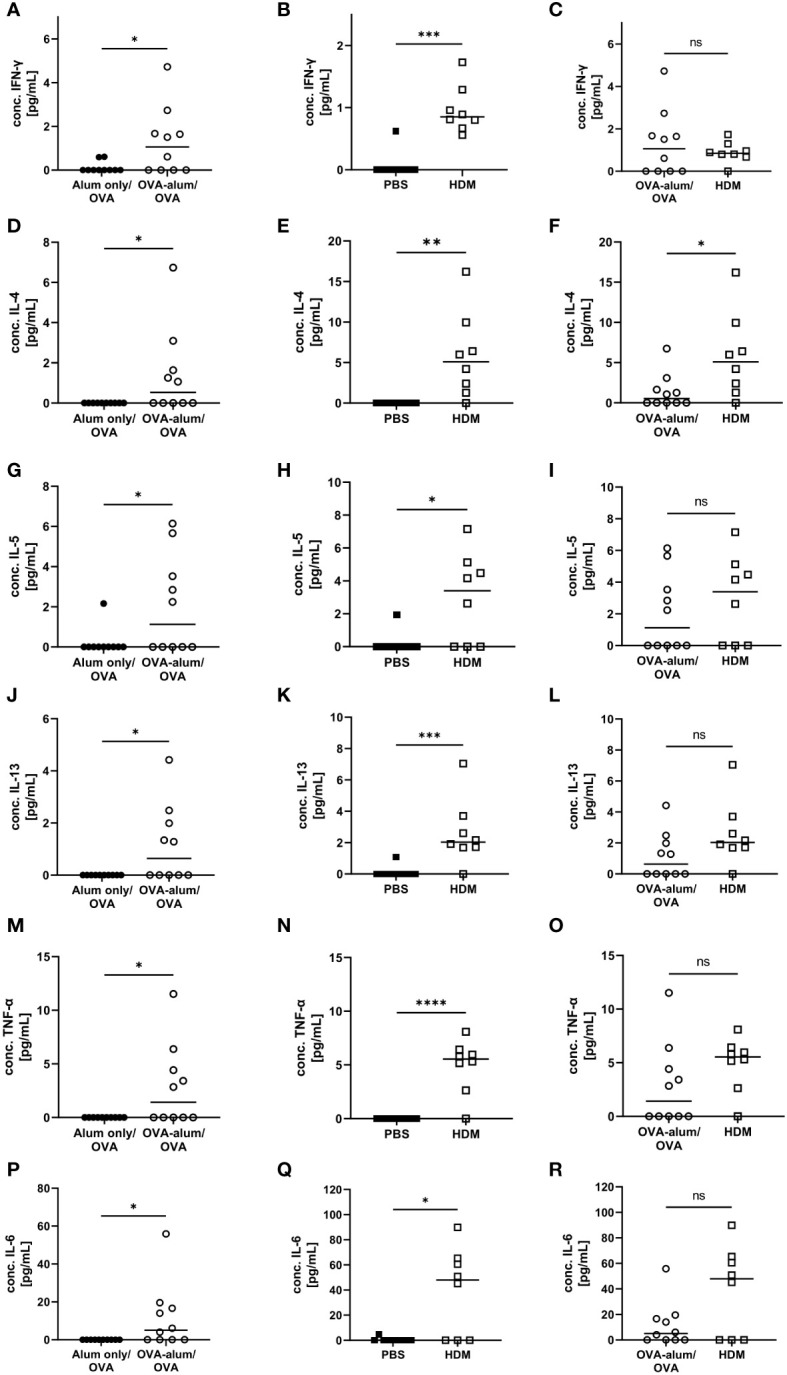
OVA- and HDM-AAI induced a significant cytokine response in the lung with model-specific characteristics. For the induction of allergic airway inflammation (AAI), mice were treated with ovalbumin (OVA; n=10) (and aluminum hydroxide (alum) for sensitization) or house dust mite extract (HDM; n=8) as described in materials and methods. Control mice were mock-sensitized with alum only (OVA-AAI; n=10) or treated with PBS only (HDM-AAI; n=9). BAL was analyzed for IFN-γ **(A, B)**, IL-4 **(D, E)**, IL-5 **(G, H)**, IL-13 **(J, K)**, TNF-α **(M, N)** and IL-6 **(P, Q)** and compared between models **(C, F, I, L, O, R)**. Data are shown for individual mice together with the group median. BAL samples were collected in three independent experiments. *p < 0.05, **p < 0.01, ***p < 0.005, ****p < 0.0001, ns, not significant.

Cellular responses in respiratory inflammation are associated with the release of cytokines. For further insight into potential differences in the phenotype of inflammation in the two models of AAI, we assessed respiratory cytokine levels. The Th1 cytokine IFN-γ was significantly elevated in OVA-AAI and HDM-AAI as compared to the control group (**Figures 3A, B**). The difference in IFN-γ between the models did not reach statistical significance (**Figure 3C**). The Th-2 cytokines IL-4, IL-5 and IL-13 were significantly increased in both OVA-AAI and HDM-AAI as compared to the respective control groups (**Figures 3D, E, G, H, J, K**). In all cases, mean cytokine levels were higher in HDM-AAI as compared to OVA-AAI (**Figures 3F, I, L**), reaching statistical significance in case of IL-4. Also the respiratory concentrations of the pro-inflammatory cytokines TNF-α and IL-6 were clearly and significantly increased in both AAI models as compared to controls (**Figures 3M, N, P, Q**). Again, there was a stronger, however not statistically significant, increase in HDM-AAI as compared to OVA-AAI (**Figures 3O, R**).

**Figure 4:**
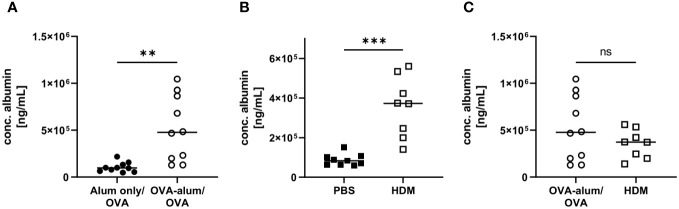
OVA-AAI and HDM-AAI led to a significant increase in the concentration of serum albumin in bronchoalveolar lavage (BAL). For the induction of allergic airway inflammation (AAI), mice were treated with ovalbumin (OVA; n=10) (and aluminum hydroxide (alum) for sensitization) or house dust mite extract (HDM; n=8) as described in materials and methods. Control mice were mock-sensitized with alum only (OVA-AAI; n=10) or treated with PBS only (HDM-AAI; n=9). Concentrations of serum albumin in BAL were determined by ELISA for OVA-AAI **(A)** and HDM-AAI **(B)** and levels were compared in **(C)**. Data compiled from at least three independent experiments are shown for individual mice with the median. **p < 0.01, ***p < 0.005, ns = not significant.

Respiratory infections and inflammatory diseases, such as asthma, can lead to reduced pulmonary function and increased capillary permeability, causing serum albumin to leak from the circulation into tissue fluid or to escape into the alveolar space (23, 24). In both AAI models, we indeed observed a significant increase in the concentration of albumin in the BAL as compared to the control groups (**Figures 4A, B**). Comparing both models, the BAL albumin concentration was marginally, however not significantly, reduced in HDM-AAI compared to OVA-AAI (**Figure 4C**).

Taken together, we detected hallmark features of AAI with respect to absolute cell numbers isolated from the lungs, eosinophil and neutrophil accumulation (for gating see **Supplementary Material**, **Supplementary Figure 2**), IgE-production and respiratory cytokine and serum albumin concentrations in both models. These parameters at times significantly varied in intensity between OVA-AAI and HDM-AAI. The direction of these model-specific differences however was not uniform, with by trend higher cell numbers and cytokine levels, significantly increased BAL IL-4 and at the same time significantly reduced serum IgE levels in HDM-AAI as compared to OVA-AAI.

**Figure 5:**
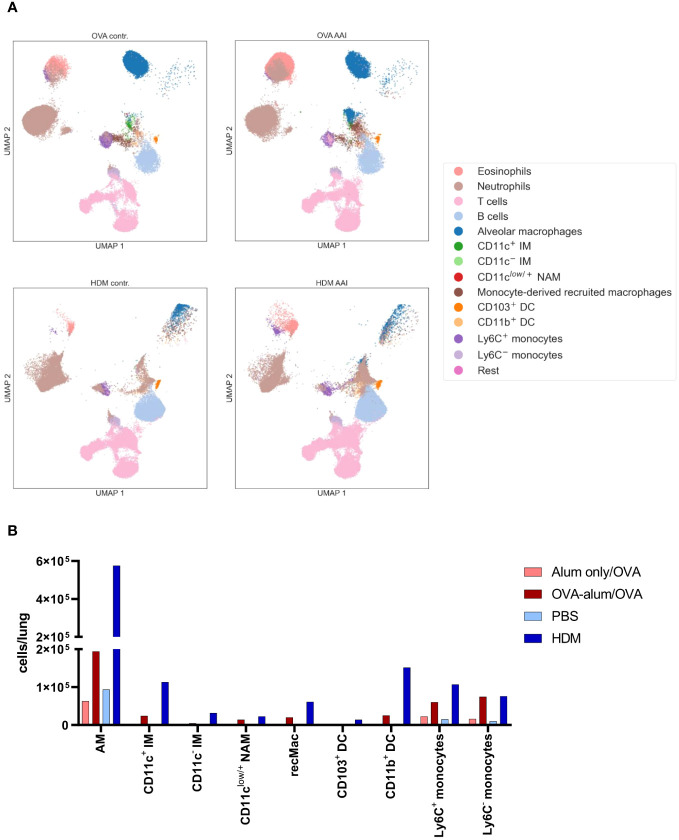
OVA-AAI and HDM-AAI showed substantial differences in the accumulation of specific immune cell subsets in the lung. For the induction of allergic airway inflammation (AAI), mice were treated with ovalbumin (OVA; n=10) (and aluminum hydroxide (alum) for sensitization) or house dust mite extract (HDM; n=8) as described in materials and methods. Control mice were mock-sensitized with alum only (OVA-AAI; n=10) or treated with PBS only (HDM-AAI; n=9). Clustering of immune cell subsets was performed by UMAP and cell subsets were identified and color-coded based on manual gating. Representative UMAP plots **(A)**. Overview of median absolute numbers of the indicated immune cells compiled from all experiments **(B)**. Individual data for these populations are shown in **Figures 6**-**11**.

For an overview of the involved immune cells, manually gated populations from FACS data were used for UMAP embedding of the analyzed cell subsets (**Figure 5A**). UMAP plotting revealed substantial differences between the AAI models compared to the corresponding controls. Absolute cell numbers of different macrophage, DC and monocyte subsets showed a model-specific accumulation in the lung (**Figure 5B**).

### 3.2 AAI distinctly affects several lung macrophage subsets with model-specific characteristics

**Figure 6:**
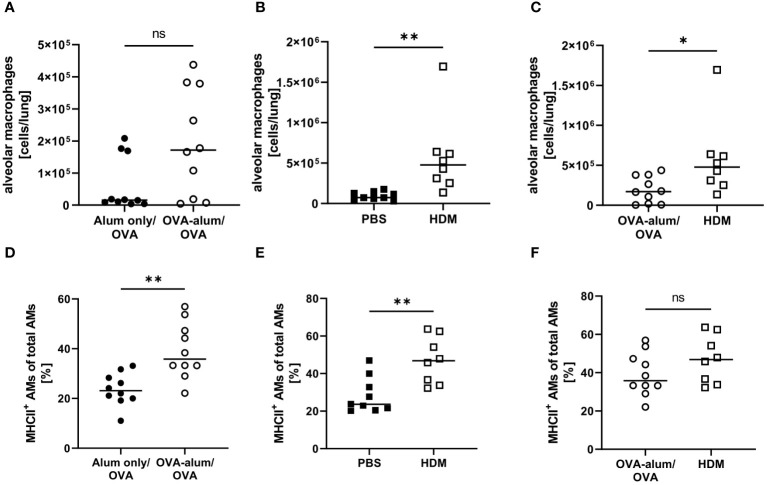
OVA- and HDM-AAI led to a model-specific accumulation of alveolar macrophages (AMs) with increased MHCII expression in the lung. For the induction of allergic airway inflammation (AAI), mice were treated with ovalbumin (OVA; n=10) (and aluminum hydroxide (alum) for sensitization) or house dust mite extract (HDM; n=8) as described in materials and methods. Control mice were mock-sensitized with alum only (OVA-AAI; n=10) or treated with PBS only (HDM-AAI; n=9). Lung leukocytes were analyzed for alveolar macrophage (AM) numbers **(A-C)** and the frequency of MHCII^+^ AM (out of all AM) **(D-F)**. Data compiled from at least three independent experiments are shown for individual mice with the median. *p < 0.05, **p < 0.01, ns, not significant.

We detected substantial numbers of AMs within the analyzed lung leukocyte pool (see **Supplementary Material**, **Supplementary Figure 2**). While AMs did accumulate to some extent in OVA-AAI, they were not significantly elevated as compared to the control group (**Figure 6A**). In HDM-AAI, AMs revealed a stronger and significant increase (**Figure 6B**) and the mean AM count in HDM-AAI was significantly increased as compared to OVA-AAI (3-fold) (**Figure 6C**). Despite the difference in AM numbers between the models, the frequency of MHCII^+^ cells within the AM pool significantly increased to similar extents in OVA-AAI and HDM-AAI as compared to the controls (**Figures 6D, E**).

**Figure 7:**
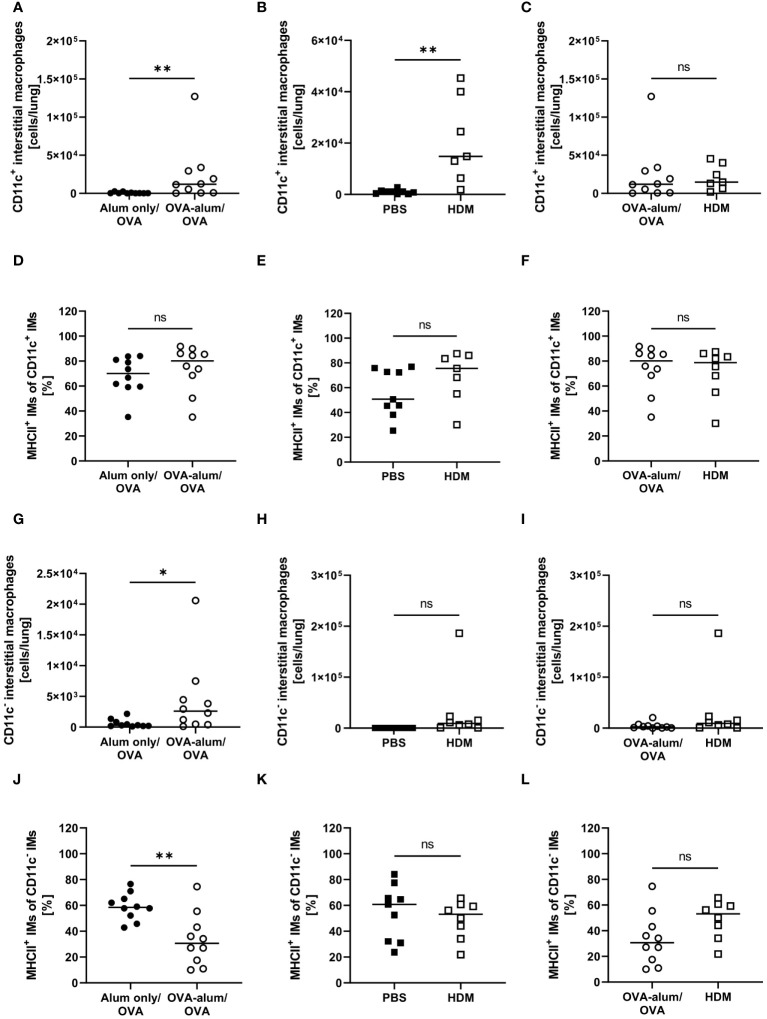
OVA- and HDM-AAI led to the accumulation of CD11c^+^ and/or CD11c^-^ interstitial macrophage (IM) subsets with model-specific effects on cell numbers and the frequency of MHCII expression. For the induction of allergic airway inflammation (AAI), mice were treated with ovalbumin (OVA; n=10) (and aluminum hydroxide (alum) for sensitization) or house dust mite extract (HDM; n=8) as described in materials and methods. Control mice were mock-sensitized with alum only (OVA-AAI; n=10) or treated with PBS only (HDM-AAI; n=9). Lung leukocytes were analyzed for absolute numbers of CD11c^+^ IMs (**A, B** (n=7 in HDM-AAI)) and the frequency of their MHCII expression (**D, E** (n=7 in HDM-AAI)), absolute numbers of CD11c^-^ IMs **(G, H)** and the frequency of their MHCII expression **(J, K)**. Results were compared between OVA-AAI and HDM-AAI **(C, F, I, L)**. Data compiled from at least three independent experiments are shown for individual mice with the median. *p < 0.05, **p < 0.01, ns, not significant.

With respect to IMs, we differentiated between CD11c^+^ and CD11c^-^ IMs (25, 26). We detected a significant increase of CD11c^+^ IMs in OVA-AAI and HDM-AAI and the number of IMs did not differ between the two models of AAI (**Figures 7A–C**). In contrast to AMs, significantly altered frequencies of MHCII^+^ CD11c^+^ IMs were neither observed in OVA-AAI nor HDM-AAI (**Figures 7C–F**). As opposed to CD11c^+^ IMs, CD11c^-^ IMs were significantly elevated only following the induction of OVA-AAI but not HDM-AAI (**Figures 7G, H**). There was however no significant difference in the number of CD11c^-^ IMs between the models (**Figure 7I**). The frequency of MHCII^+^ CD11c^-^ IMs significantly decreased in OVA-AAI and slightly decreased in HDM-AAI (**Figures 7J, K**). Even though not statistically significant, the MHCII expression frequency of CD11c^-^ IM was higher in HDM-AAI as compared to OVA-AAI (**Figure 7L**).

**Figure 8:**
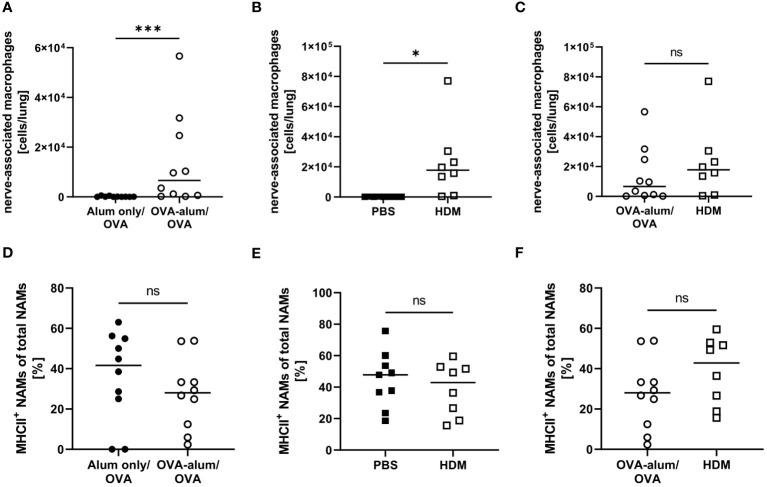
OVA- and HDM-AAI led to the accumulation of nerve-associated macrophages (NAMs) in the lung but did not affect the frequency of MHCII expression. For the induction of allergic airway inflammation (AAI), mice were treated with ovalbumin (OVA; n=10) (and aluminum hydroxide (alum) for sensitization) or house dust mite extract (HDM; n=8) as described in materials and methods. Control mice were mock-sensitized with alum only (OVA-AAI; n=10) or treated with PBS only (HDM-AAI; n=9). Lung leukocytes were analyzed for absolute numbers of NAMs **(A, B)** and the frequency of their MHCII expression **(D, E)**. Results were compared between models **(C, F)**. Data compiled from at least three independent experiments are shown for individual mice with the median. *p < 0.05, ***p < 0.005, ns, not significant.

Little is known with respect to the role of NAMs in AAI and we included this recently described, specialized IM subset in our analyses. In the lung, we observed a significant increase of NAMs in OVA-AAI as well as HDM-AAI (**Figures 8A, B**). The median NAM number was higher in HDM-AAI as compared to OVA-AAI, even though this increase did not reach statistical significance. As opposed to other macrophage subsets, the frequency of MHCII^+^ NAMs was not altered in either model of AAI as compared to the respective controls or between models (**Figures 8C, F**).

**Figure 9:**
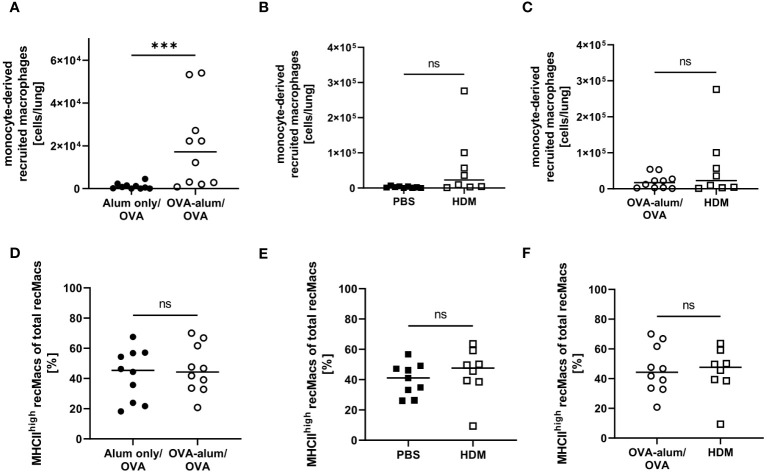
OVA-AAI but not HDM-AAI led to a significant increase of recruited macrophages (recMacs) in the lung but did not affect the frequency of MHCII^high^ recMacs. For the induction of allergic airway inflammation (AAI), mice were treated with ovalbumin (OVA; n=10) (and aluminum hydroxide (alum) for sensitization) or house dust mite extract (HDM; n=8) as described in materials and methods. Control mice were mock-sensitized with alum only (OVA-AAI; n=10) or treated with PBS only (HDM-AAI; n=9). Lung leukocytes were analyzed for absolute numbers of recMacs **(A, B)** and the frequency of high MHCII expression **(D, E)**. Results were compared between models **(C, F)**. Data compiled from at least three independent experiments are shown for individual mice with the median. ***p < 0.005, ns, not significant.

During inflammation, also monocyte-derived recruited macrophages (recMacs) accumulate in the lung. RecMacs are CD11b^+^, MHCII^+^ and CD64^+^ but do not co-express MerTK and CD64, distinguishing them from AM and IM (see gating in **Supplementary Material**, **Supplementary Figure 2**) (27). RecMacs identified by our gating express CD11c and MHCII-expression discriminates them from monocytes. In OVA-AAI but not HDM-AAI, we observed a significant increase in the number of monocyte-derived recMacs in the lung as compared to the respective control group (**Figures 9A, B**), without a significant difference between both AAI models (**Figure 9C**). The median frequency of MHCII^high^ recMacs was not altered in AAI as compared to controls (**Figures 9D-F**).

Taken together, we observed substantial alterations to the respiratory macrophage compartment in AAI that were not only subset- but also model-specific. For two of the analyzed macrophage populations, CD11c^+^ IMs and NAMs, we observed a significant increase in numbers in the lung in OVA-AAI and HDM-AAI. For AMs, we observed this significant increase only in HDM-AAI, resulting in a significant difference for AM comparing both models. The numbers of CD11c^-^ IMs and recMacs were only significantly increased in OVA-AAI as compared to HDM-AAI. A significant effect of AAI on the frequency of MHCII expression within macrophage subsets was only detected for AM and was not significantly altered between HDM- and OVA-AAI.

### 3.3 OVA-AAI and HDM-AAI significantly affect the lung DC compartment with subset-specific effects on the activation status

Given their central role in inducing and shaping allergic responses in the airways, we furthermore focused on the numbers and the activation status of different DC populations in the lungs in OVA-AAI and HDM-AAI.

**Figure 10:**
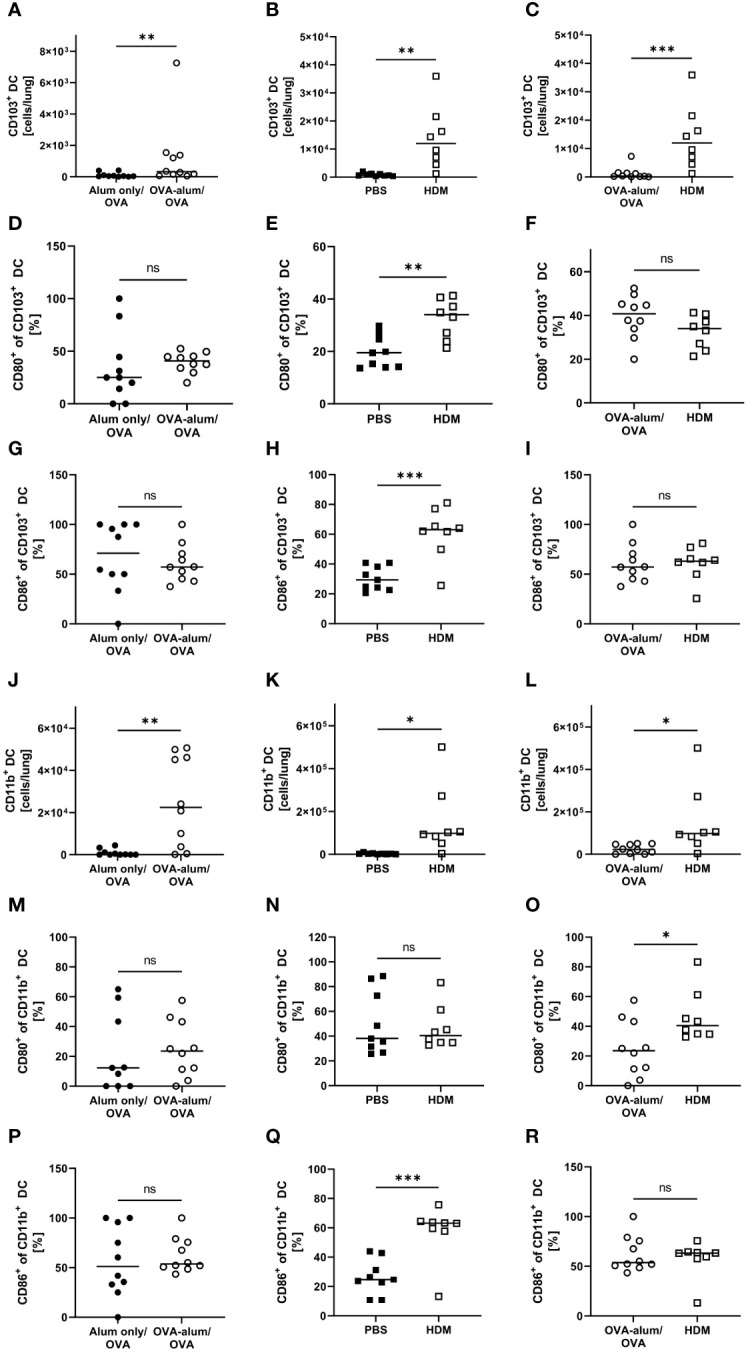
OVA- and HDM-AAI led to an accumulation of CD103^+^ and CD11b^+^ dendritic cells (DC) in the lung with subset- and model model-specific effects on cell numbers, CD80^-^ and CD86^-^expression. For the induction of allergic airway inflammation (AAI), mice were treated with ovalbumin (OVA; n=10) (and aluminum hydroxide (alum) for sensitization) or house dust mite extract (HDM; n=8) as described in materials and methods. Control mice were mock-sensitized with alum only (OVA-AAI; n=10) or treated with PBS only (HDM-AAI; n=9). Lung leukocytes were analyzed and compared between models for cell numbers of CD103^+^ DC **(A-C)** and the frequency of their expression of the activation markers CD80 **(D-F)** and CD86 **(G-I)**. Furthermore, cell numbers of CD11b^+^ DC **(J-L)** and the frequency of their expression of the activation markers CD80 **(M-O)** and CD86 **(P-R)** were analyzed and compared between models. Data compiled from at least three independent experiments are shown for individual mice with the median. *p < 0.05, **p < 0.01, ***p < 0.005, ns, not significant.

Lung CD103^+^ DCs were significantly elevated in both AAI models as compared to the respective controls (**Figures 10A, B**). Comparing both models revealed the number of CD103^+^ DCs to be strongly and significantly increased in HDM- as compared to OVA-AAI (11-fold; **Figure 10C**). We furthermore observed a significant increase of the expression of the activation markers CD80 and CD86 in terms of the frequency of marker-expressing CD103^+^ DCs only in HDM-AAI but not OVA-AAI as compared to their control groups (**Figures 10D, E, G, H**). In OVA-AAI, we observed a slight increase in CD80^+^ CD103^+^ DCs, although this increase was not statistically significant (**Figure 10D**). The frequency of CD80^+^ and CD86^+^ CD103^+^ DCs was unchanged between OVA-AAI and HDM-AAI (**Figures 10F, I**).

As CD103^+^ DCs, also lung CD11b^+^ DCs showed significantly increased numbers in both models of AAI (**Figures 10J, K**). Also the overall number of CD11b^+^ DCs was significantly higher in HDM-AAI as compared to OVA-AAI (6-fold; **Figure 10L**) which was consistent with the results for CD103^+^ DCs. While the slight increase in the frequency of CD11b^+^ DC expressing the activation marker CD80 was not significant in both AAI models as compared to their controls (**Figures 10M, N**), the frequency of CD86-expressing CD11b^+^ DCs was clearly and significant elevated in HDM-AAI but not in OVA-AAI (**Figures 10P, Q**). Furthermore, the frequency of CD80^+^ CD11b^+^ DC was significantly higher in HDM-AAI as compared to OVA-treated mice (**Figure 10O**) while the percentage of CD86^+^ CD11b^+^ DCs was unchanged between the models (**Figure 10R**).

Taken together, a clear increase of CD103^+^ DCs and CD11b^+^ DCs was observed after induction of AAI either with OVA or HDM. For both DC populations, this increase was significantly higher in HDM-AAI compared to OVA-AAI. With respect to the activation status of both DC populations, we observed model- as well as marker-specific results. Whereas in CD103^+^ DCs, activation comprised CD80- and CD86-expression and was restricted to HDM-AAI, activation was restricted to CD86-expression in CD11b^+^ DCs, also detectable exclusively in HDM-AAI.

### 3.4 Monocyte numbers in the lung are affected in AAI

**Figure 11:**
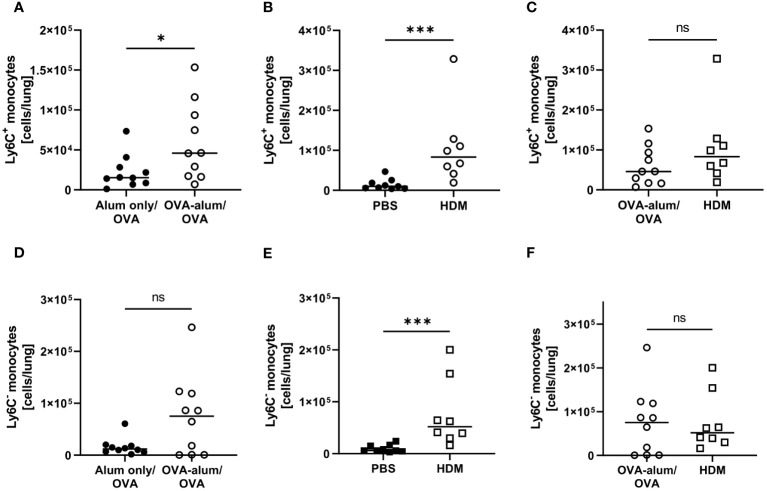
OVA- and HDM-AAI led to a significant increase of Ly6C^+^ and Ly6C^-^ monocytes in the lung with model-specific differences in Ly6C- monocytes. For the induction of allergic airway inflammation (AAI), mice were treated with ovalbumin (OVA; n=10) (and aluminum hydroxide (alum) for sensitization) or house dust mite extract (HDM; n=8) as described in materials and methods. Control mice were mock-sensitized with alum only (OVA-AAI; n=10) or treated with PBS only (HDM-AAI; n=9). Lung leukocytes were analyzed for numbers of Ly6C^+^ monocytes **(A-C)** and Ly6C^-^ monocytes **(D-F)** and compared between the models. Data compiled from at least three independent experiments are shown for individual mice with the median. *p < 0.05, ***p < 0.005, ns, not significant.

As monocytes can differentiate into populations of macrophages and monocyte-derived DC in the tissue and as we had detected clear effects on the respiratory myeloid cell compartment in AAI, we further analyzed two distinct monocyte populations within the leukocytes isolated from the lung (Ly6C^+^ and Ly6C^-^). Indeed, we observed a significant increase in lung Ly6C^+^ monocytes in both models of AAI, whereas Ly6C^-^ monocytes were significantly increased only in HDM-AAI (**Figures 11A, B, D, E**). The median absolute number of Ly6C^+^ monocytes was slightly higher in HDM-AAI as compared to OVA-AAI (1.8-fold), but this increase did not reach statistical significance (**Figure 11C**). In contrast, for Ly6C^-^ monocytes we observed slightly higher numbers (1.3-fold increase) in OVA-AAI as compared to HDM-AAI albeit with no statistical significance (**Figure 11F**).

In summary, we show that next to a significant increase in lung Ly6C^+^ monocytes irrespective of the analyzed model, also monocyte accumulation partly depended on the experimental model.

## 4 Discussion

With our study, we provide a comprehensive analysis of key inflammatory cells as well as macrophage and DC populations in experimentally induced AAI. While DC play documented roles in AAI, the exact contributions of the different subsets to the induction and maintenance of inflammation remain elusive in many points. In recent years, our understanding of lung macrophages in lung health and disease has substantially increased and novel macrophage populations with distinct developmental origins, phenotypes and functions have been identified. Mouse models are widely used in this context. Based on experimental animal studies, mechanistic conclusions are drawn that may be lost using a different model. In our analyses, we included macrophage and DC subsets not typically resolved in conventional flow cytometry. High-parameter spectral flow cytometry allows for staining a high number of cellular markers and thereby for the acquisition of comprehensive phenotypic data in heterogeneous cell suspensions with a high sensitivity to detect rare populations. Further, it allows handling of AF signals to increase the resolution in flow cytometric analyses of highly autofluorescent cells such as tissue macrophages (20). For comparison, gating of lung leukocytes without AF handling is shown in **Supplementary Figure 3**. Different approaches are available for experimental AAI and detailed knowledge regarding the induced inflammatory response is essential for choosing a suited model for specific research questions. We analyzed distinct macrophage, DC and monocyte populations next to key inflammatory cells such as eosinophils and neutrophils in the lung in two models of experimental AAI. Here, we focused on peripheral sensitization with a model-allergen together with an adjuvant (OVA-AAI) and respiratory sensitization with the natural allergen HDM (HDM-AAI). Overall, our study underlines the importance to consider model-specific effects in the conclusions drawn from experimental animal studies, particularly with respect to the distinct involvement of macrophages and DC.

Our results demonstrate several subsets of macrophages, i.e. AM, CD11c^+^ and CD11c^-^ IM, NAM and recMac, to be significantly involved in experimental AAI. This likewise applied to CD103^+^ DC and CD11b^+^ DC as well as Ly6C^+^ and Ly6C^-^ monocytes. The detected changes in the macrophage, DC and monocyte compartments were accompanied by classical effectors such as lung eosinophils and neutrophils as well as elevated Th1, Th2 and pro-inflammatory cytokines in the BAL and elevated systemic IgE levels. Of note, our antibody panel focusing on myeloid cell subsets also allowed analysis of pan T cell numbers (28) and B cells (for gating see **Supplementary Figure 2**). T cell numbers in the lung were elevated in OVA-AAI (without statistical significance; p = 0.3930) and HDM-AAI (p = <0.0001) with a significant increase in HDM-AAI over OVA-AAI (**Supplementary Figure 4**). The analysis of lung B cells showed similar results as for T cells with elevated numbers in OVA-AAI (without statistical significance; p = 0.0753) and HDM-AAI (p = <0.0001) and a significant increase in HDM-AAI over OVA-AAI (**Supplementary Figure 5**).

Pulmonary macrophages are highly plastic and respond to the local microenvironment by adopting dynamic, multidimensional phenotypic profiles (29). During homeostasis, two main subsets of macrophages coexist in the lung and of these, AM are the major embryonically derived population found in the alveolar spaces. Further, IMs, thought to derive from blood monocytes, reside within the lung parenchyma and comprise phenotypically distinct subpopulations (8). However, also monocytes can differentiate into AM (30). We have analyzed cells isolated from lavaged lung tissue. Even though AM populate the airspaces, we detected substantial AM numbers in the lung that were specifically increased in HDM-AAI. Presumably, these AM also represent remaining AM from remaining cells after lavage (31). The frequency of MHCII expression on the detected AM increased in AAI, which is in line with MHCII expression of inflammatory (M2) rather than homeostatic (M1) AM (32) and further demonstrates plasticity of the AM pool in AAI, especially when mediated by HDM.

Even though likewise yolk-sac derived, NAMs are morphologically and transcriptionally distinct from AMs suggesting a specialized role. They have been described to expand and to regulate inflammatory responses during acute Influenza A virus (IAV) infection (9). While there is increasing evidence for neuro-immune crosstalk in allergic asthma (33), a possible role for these cells in regulating AAI has not been addressed. In our analyses, we indeed observed increased numbers of NAMs in the lungs in AAI. While transcription of MHCII genes in NAM has been described (9), the frequency of MHCII^+^ NAM was not affected in AAI. We have identified NAMs according to their described surface marker expression and their true nature will need to be confirmed by imaging *in situ* in the lung tissue upon AAI. Also, future analyses will have to reveal potential functional roles of increased numbers of NAM in the lung in regulating AAI.

IMs can be divided into several subpopulations such as CD11c^+^ and CD11c^-^ IMs (9, 25, 26). Further, monocytes developing into macrophages are recruited to the lung under inflammatory conditions. The accumulation pattern of these macrophage subsets in the lung was partly model-specific. CD11c^+^ IMs significantly increased in OVA-AAI and HDM-AAI, while CD11c^-^ IMs and recMacs were significantly increased only in OVA-AAI. The frequency of MHCII expression on these IM was generally higher than that on AM, but it was not (recMac) or only marginally (CD11c^+^ and CD11c^-^ IM) affected by AAI. A role for MHCII on lung IMs in providing a niche for tissue-resident CD4^+^ T cells has been suggested, but its precise function in this context remains unclear (34). The expression of MHCII and other costimulatory molecules is essential for the activation of CD4^+^ T cells (35). Professional antigen-presentation to T cells is mainly attributed to DC and AM have originally been ascribed rather regulatory roles (36). So far, relatively little is known with respect to their functional roles in the induction and maintenance of AAI. They have been shown to support the induction of regulatory T cells (37) but also to promote cytokine production by allergen-specific Th2 cells, involving the expression of CD80 and CD86 (38, 39). In the AM population we detected in our analyses, the frequency of CD80^+^ and CD86^+^ AM was reduced in both OVA- as well as HDM-AAI as compared to the respective controls or remained unchanged. The mean fluorescence intensity (MFI) of the CD80 staining on CD80^+^ AM was nearly unchanged in OVA- and HDM-AAI as compared to controls (1.2 and 1.3-fold, respectively). In contrast, CD86 expression (MFI) on CD86^+^ AM was increased 3.1-fold in HDM-AAI as compared to 1.4-fold in OVA-AAI (p = 0.007) (**Supplementary Figure 6**). Indeed, Balbo et al. reported CD86, but not CD80 to increase on AM following allergen challenge in patients allergic to *Dermatophagoides*. Since we detected similar results in HDM- but not OVA-AAI, this possibly displays an HDM-specific mechanism in allergic asthma. However, clearly more detailed analyses will be needed to further explore on this issue und clarify its relevance in the differences observed between OVA- and HDM-AAI. Further, resident macrophages associated to the bronchi (BAM) have recently been described to capture and present antigens and activate Th2 cells in the lung (40). These BAM are CD11c^+^ IM characterized by high CX3CR1 and MHCII expression. While our spectral flow cytometry antibody panel allows their identification with respect to the expression of these markers, our studies at this point have not included imaging of lung tissue to validate their identity as BAM. Therefore, further studies combining spectral flow cytometry with imaging and molecular approaches such as *in situ* transcriptomics will be required to parse out the functional involvement of the different macrophage subsets in AAI. Generally, our knowledge with respect to the complex functions and plasticity of pulmonary macrophages in AAI remains incomplete (27). While our approach allowed the simultaneous identification and quantification of a substantial number of previously described macrophage subtypes in AAI, it provides only limited insight into their function beyond their accumulation and alterations in MHC-expression. Nevertheless, we believe our study provides a valuable basis for future mechanistic studies that, according to our data, should differentiate between models of AAI.

DCs are a complex and heterogeneous innate immune cell population that localizes in most tissues in the steady state. Here, DCs recognize and respond to pathogen-associated and danger-associated signals. In mice, the lung parenchyma contains two conventional DC (cDC) populations that accumulate near the small airway epithelia: CD103^+^ and CD11b^+^ DC (13). Both, CD103^+^ and CD11b^+^ DCs, have been recognized as critical regulators of allergen-driven immune responses in the lung (14, 41). Plantinga et al. identified CD11b^+^ cDC as the main subset inducing Th2 cell-mediated immunity in HDM-mediated experimental allergic airway inflammation (41). While the contribution of CD11b^+^ cDCs to AAI is widely accepted, the function of CD103^+^ remains controversially discussed (15). Overall, we detected significantly increased numbers of DC of both populations in the lung independent of the AAI model, which is contrast to reports of unchanged numbers of CD103^+^ DC in mouse models of AAI (15). Significantly elevated numbers of CD103^+^ as well as CD11b^+^ DC in HDM-AAI as compared to OVA-AAI however point at a stronger involvement of DC in HDM-mediated inflammation. This is underscored by significantly elevated (as compared to controls) frequencies of CD80^+^ and CD86^+^ CD103^+^ DC as well as CD86^+^ CD11b^+^ DC exclusively in HDM, but not OVA-AAI. Further, this finding demonstrates not only a numeric increase in DC numbers but also significantly increased functional activation specifically in this model of AAI. Given the central role of DC in driving Th2-inflammation in AAI (41), their increased number and activation are well in line with the significantly elevated levels of IL-4 we detected in the BAL in HDM-AAI as compared to OVA-AAI. Comparing the treatment regimen of the two AAI models (see **Figure 1**), increased activation and accumulation of DC (and other cells) in the lung in HDM-AAI could well result from the extended period of airway challenges in this model as compared to OVA-AAI. While in HDM-AAI allergen was applied in weekly intervals over two weeks prior to analysis, respiratory allergen challenges in OVA-AAI only occurred for three consecutive days before analysis. Presumably, upstream involvement of the respiratory epithelium fundamentally differs between OVA-AAI and HDM-AAI due to the extended time-span of respiratory allergen-treatments, protease activity of the allergen and local innate immune stimulation in HDM-AAI (18, 42, 43), which is in turn likely to contribute to model-specific characteristics in inflammation. In this context, the comparable serum albumin levels detected in the BAL for both AAI models appear even more remarkable.

In addition to cDC, monocyte-derived moDC occur in the murine lung and add to AAI mainly by cytokine production (41). Further, AM and other macrophage subsets develop from monocytes recruited to the lung in the course of inflammation as described above. To gain insight into their involvement in AAI in the context of macrophage and DC accumulation, we analyzed monocyte numbers from our spectral flow cytometry data. Mouse monocyte subsets are characterized by differential expression of the inflammatory monocyte marker Ly6C. Inflammatory, Ly6C^+^ monocytes are found in higher numbers in asthmatic patients and contribute to increased inflammation in mice with AAI (6, 44, 45). In line with this, we found Ly6C^+^ monocytes in the lungs to be significantly elevated in both OVA- and HDM-AAI. Significant accumulation of Ly6C^-^ monocytes was only detected in HDM-AAI. These results demonstrate that AAI in these models harbors distinct characteristics also with respect to the accumulation of monocytes in the lungs and they further support our view that there is not generally elevated inflammation in HDM-AAI as compared to OVA-AAI. According to a recent report however, it cannot be excluded, these monocytes are of vascular rather than parenchymal origin, complicating interpretation of the data (31).

**Figure 12:**
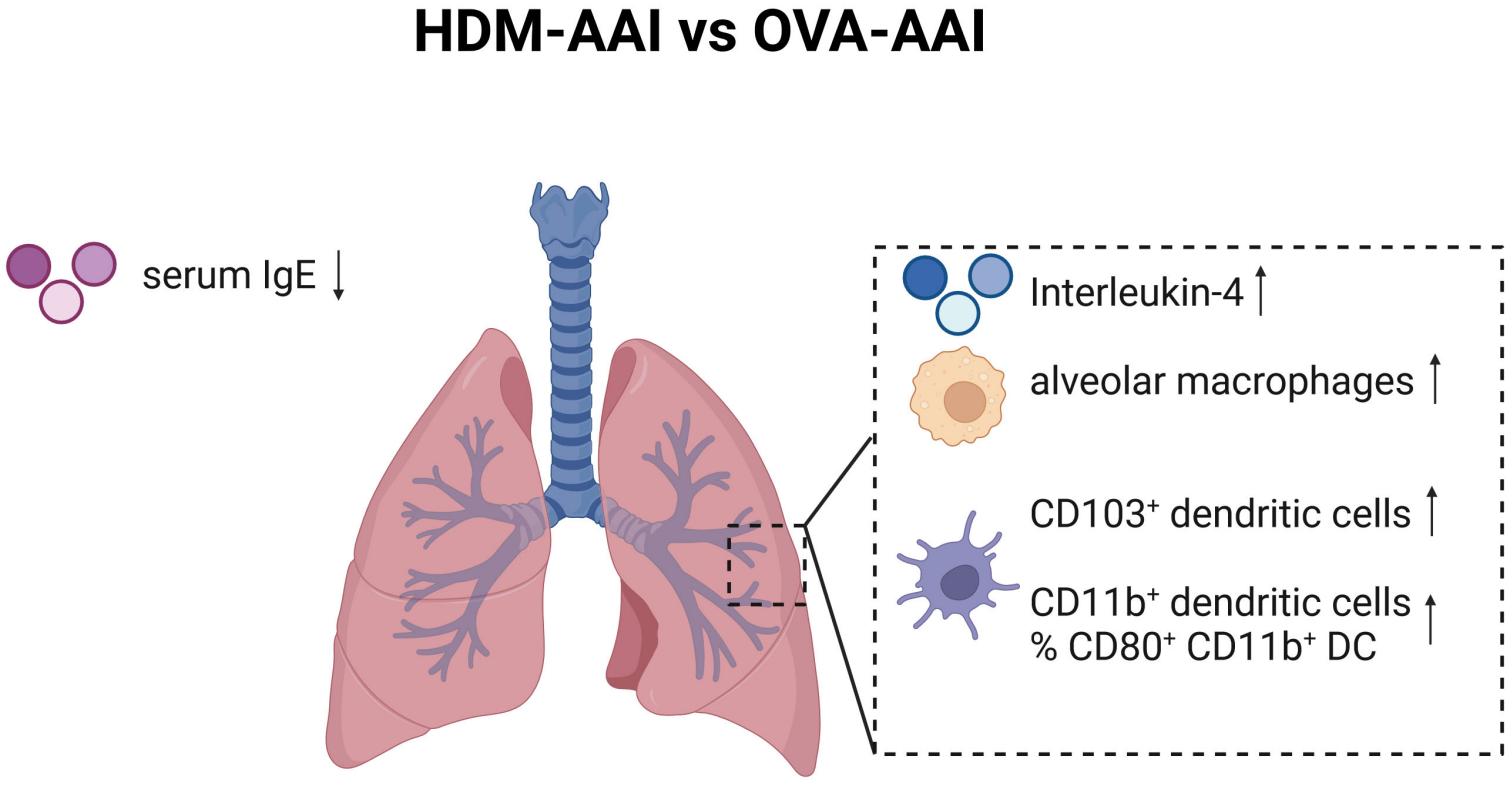
Graphical summary of significant changes between HDM-AAI and OVA-AAI. Significant increases comparing HDM-AAI to OVA-AAI are displayed with an upward arrow, significant decreases with a downward arrow. IMs, interstitial macrophages; DC, dendritic cells; IgE, immunoglobulin E. Created in BioRender.com.

Our study focused on the simultaneous detection of multiple myeloid cells of the innate immune system in two distinct models of AAI by spectral flow cytometry. We included major macrophage and DC subsets known to be involved in regulating pulmonary immune responses next to some that had not been studied in AAI (e.g. NAMs) or for which the involvement and functions in AAI remain controversial (e.g. CD103^+^ DC). Next to an overall strong involvement of macrophages, DC and monocytes in the lung, we identified several model-specific characteristics in the response (summarized in **Figure 12**). OVA-AAI involves peripheral, adjuvanted sensitizations followed by respiratory challenges with OVA-protein alone. In HDM-AAI, a natural mixture of allergens with protease-activity is administered exclusively via the airways without an additional adjuvant. Since these models differ in more than one variable, at this point, one can only speculate whether the differences we detected result from the nature of the allergen, adjuvant-use, the route of sensitization or a combination of these factors. Further, while our results show a strong reaction of the macrophage and DC cellular compartment during experimental AAI, our analyses at this point cannot distinguish, whether the accumulation of specific subsets of these cells results from a recruitment to the inflamed tissue or their local expansion. Significant differences between OVA-AAI and HDM-AAI lay in the accumulation of AM and the accumulation as well as functional marker expression of DC. Therefore, HDM-AAI might be more suitable as compared to OVA-AAI for AM- or DC-focused questions. The overall more intense inflammatory response in our model of HDM-AAI as compared to OVA-AAI however, may make it less suitable for studying modulation of AAI, e.g. by interventions. Further, it may prove beneficial to clearly separate the induction of allergen-specific sensitization from the allergen-challenge in terms of the compartment and the timing, which is the case in OVA-AAI. Here, acute respiratory allergic responses can be studied in naïve airways of pre-sensitized hosts, while discrimination between sensitization, allergic reaction and direct airway inflammation can be difficult in HDM-AAI (46). Also, when studying the role of the respiratory epithelium e.g. for macrophage or DC responses in AAI, the fundamental differences between OVA-AAI and HDM-AAI such as peripheral pre-sensitization in OVA-AAI, protease activity of HDM acting on the epithelial barrier and the time span, over which respiratory allergen treatments are performed, need to be taken into account. From these considerations, there seems to be no definite wrong or right in choosing between models of AAI. Further, there are multiple adjustments to the protocols for both OVA-AAI and HDM-AAI that we used in our study. However, studies like ours aid this choice and highlight that the results obtained and mechanisms defined using murine models for AAI need to be interpreted in the light of these considerations and may be model-specific.

Taken together, our multi-parameter spectral flow cytometry study demonstrates a strong involvement of the lung macrophage and DC compartments during experimental AAI. We believe these data provide a valuable basis for further mechanistic studies of their functions in allergic asthma and aid researchers towards choosing suited experimental models, depending on the target population and scientific hypothesis.

## Data availability statement

The raw data supporting the conclusions of this article will be made available by the authors, without undue reservation.

## Ethics statement

The animal study was approved by Landesverwaltungsamt Sachsen-Anhalt. The study was conducted in accordance with the local legislation and institutional requirements.

## Author contributions

BC: Conceptualization, Data curation, Formal analysis, Investigation, Methodology, Visualization, Writing – original draft, Writing – review & editing. IJ: Methodology, Writing – review & editing. FS: Investigation, Writing – review & editing. AP: Investigation, Writing – review & editing. AJ: Conceptualization, Data curation, Formal analysis, Visualization, Writing – review & editing. DB: Methodology, Writing – review & editing. JS: Conceptualization, Funding acquisition, Resources, Writing – review & editing. SS-K: Conceptualization, Data curation, Investigation, Methodology, Writing – original draft, Writing – review & editing.
