# Supplementary material for: Comprehensive analysis of lung macrophages and dendritic cells in two murine models of allergic airway inflammation reveals model- and subset-specific accumulation and phenotypic alterations
Source: Front Immunol. 2024 Mar 11;15:1374670. doi: 10.3389/fimmu.2024.1374670 (PMC10961404; doi:10.3389/fimmu.2024.1374670)
Supplement: Supplementary file 1 [file DataSheet_1.docx]

Supplementary Material

**Supplementary Table 1.** Cell surface marker expression

| Population | Cell surface marker |
| --- | --- |
| Alveolar macrophages (AM) | MerTK^+^/CD64^+^/Ly6C^-^/CD11c^+^/CD169^+^/Siglec-F^+^/CD11b^-^ |
| CD11c+ interstitial macrophages (IM) | MerTK^+^/CD64^+^/Ly6C^-^/CX3CR1^+^/CD169^-^/CD11c^+^ |
| CD11c- interstitial macrophages (IM) | MerTK^+^/CD64^+^/Ly6C^-^/CX3CR1^+^/CD169^-^/CD11c^-^ |
| Nerve- and airway-associated macrophages (NAM) | MerTK^+^/CD64^+^/Ly6C^-^/CX3CR1^+^/CD169^+^/CD11c^+/low^ |
| Monocyte-derived recruited macrophages (recMac) | CD64^+^/CD11b^+^ |
| CD103+ dendritic cells (DC) | CD11c^+^/MHCII^+^/CD11b^-^/CD24^+^/CD103^+^ |
| CD11b+ dendritic cells (DC) | CD11c^+^/MHCII^+^/CD24^-^/CD103^-^/CD11b^+^ |
| Ly6C+ monocytes | SiglecF^-^/CD11c^-^/CD11b^+^/MHCII^-^/CD64^-^/Ly6C^+^ |
| Ly6C- monocytes | SiglecF^-^/CD11c^-^/CD11b^+^/MHCII^-^/CD64^-^/CX3CR1^+^/Ly6C^-^ |
| Eosinophils | Ly6G^-^/SiglecF^+^/CD11c^-^/CD64^-^ |
| Neutrophils | Ly6G^+^/CD11b^+^/CD64^-^ |
| T cells | CD11b^-^/MHCII^-^/CD24^-^ |
| B cells | CD11b^-^/MHCII^+^/CD24^low/+^ |


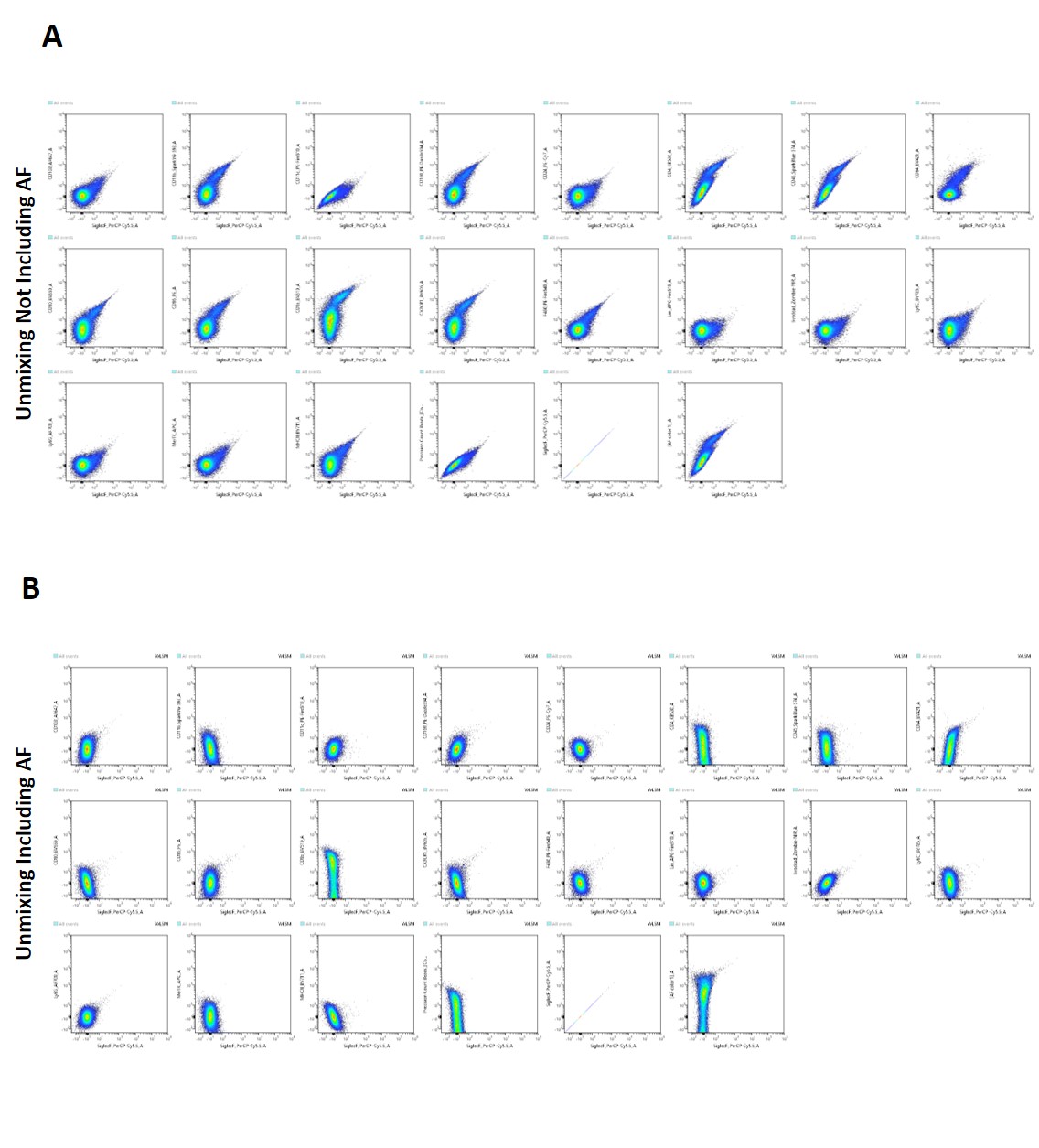

**Supplementary Figure 1.** Unmixing of spectral flow cytometry data with and without autofluorescence (AF) handling.

There is improved resolution of the unstained sample with AF included in unmixing. The unstained lung cell sample is representatively displayed in dot plots for perCP-Cy5.5 (Siglec-F) vs. every fluorophore of the staining panel. (A) shows results for unmixing applied without including the AF population in the unmixing. (B) shows results for unmixing with including the AF population, as it was performed for data analysis.


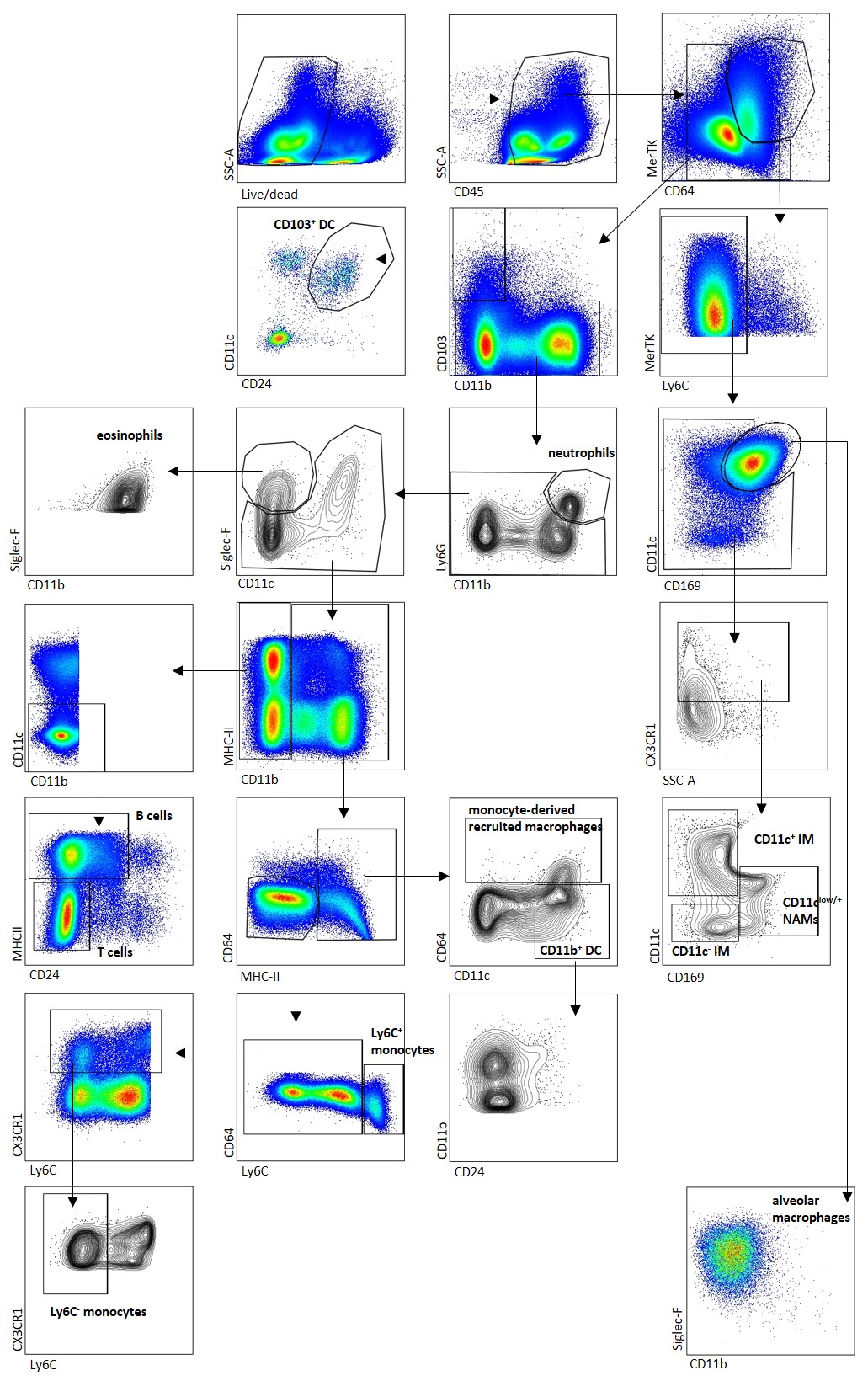


**Supplementary Figure 2.** Flow cytometry gating strategy for cells isolated from lung.
After the exclusion of douplets and dead cells, cell populations were gated as follows: Live single CD45^+^ cells were gated for MerTK^+^/CD64^+^ cells. MerTK^+^/CD64^+^ cells were further gated for Ly6C^-^ cells and these then divided into AMs (CD11c^+^/CD169^+^/SiglecF^+^/CD11b^-^) and remaining cells. From the remaining cells, CD11c^+^ IMs (CD11c^+^/CD169^-^/CX3CR1^+^), CD11c^-^ IMs (CD11c^low^/CD169^-^/CX3CR1^+^) and CD11c^low/+^ NAMs (CD11c^-/low^/CD169^high^/CX3CR1^+^) were gated. Remaining cells from the CD64/MerTK gating were further gated into CD103^+^ DC (CD11c^+^/CD11b^-^/CD103^+^/CD24^+^), neutrophils (CD11b^+^/Ly6G^+^) and eosinophils (CD11b^+^/CD11c^-^/Ly6G^-^/Siglec-F^+^). Remaining cells from the SiglecF/CD11c gating that were not eosinophils were gated for CD11b^+/high^ cells. From these, MHCII^+^ cells were gated. From the CD11b^+/high^/MHCII^+^ cells, monocyte-derived recruited macrophages (CD11b^+/high^/MHCII^+^/CD64^+^) and CD11b^+^ DC (CD11b^+/high^/MHCII^+^/CD11c^+^/CD64^-^) were gated. For monocytes, CD11b^+/high^ cells were gated for MHCII^-^~~/~~CD64^-^ cells that were further divided into Ly6C^+^ monocytes and Ly6C^-/low^ cells. Ly6C^-/low^ cells were gated for CXCR31^+^/Ly6C^-­^ monocytes. T cells were gated as CD11b^-^/CD11c^-^/CD24^-^/MHCII^-^ and B cells were gated as CD11b^-^/CD11c^-^/MHCII^+^/CD24^low/+^. Displayed here are concatenated samples of all analyzed groups. Cells were measured at Sony ID7000 spectral analyzer.

**
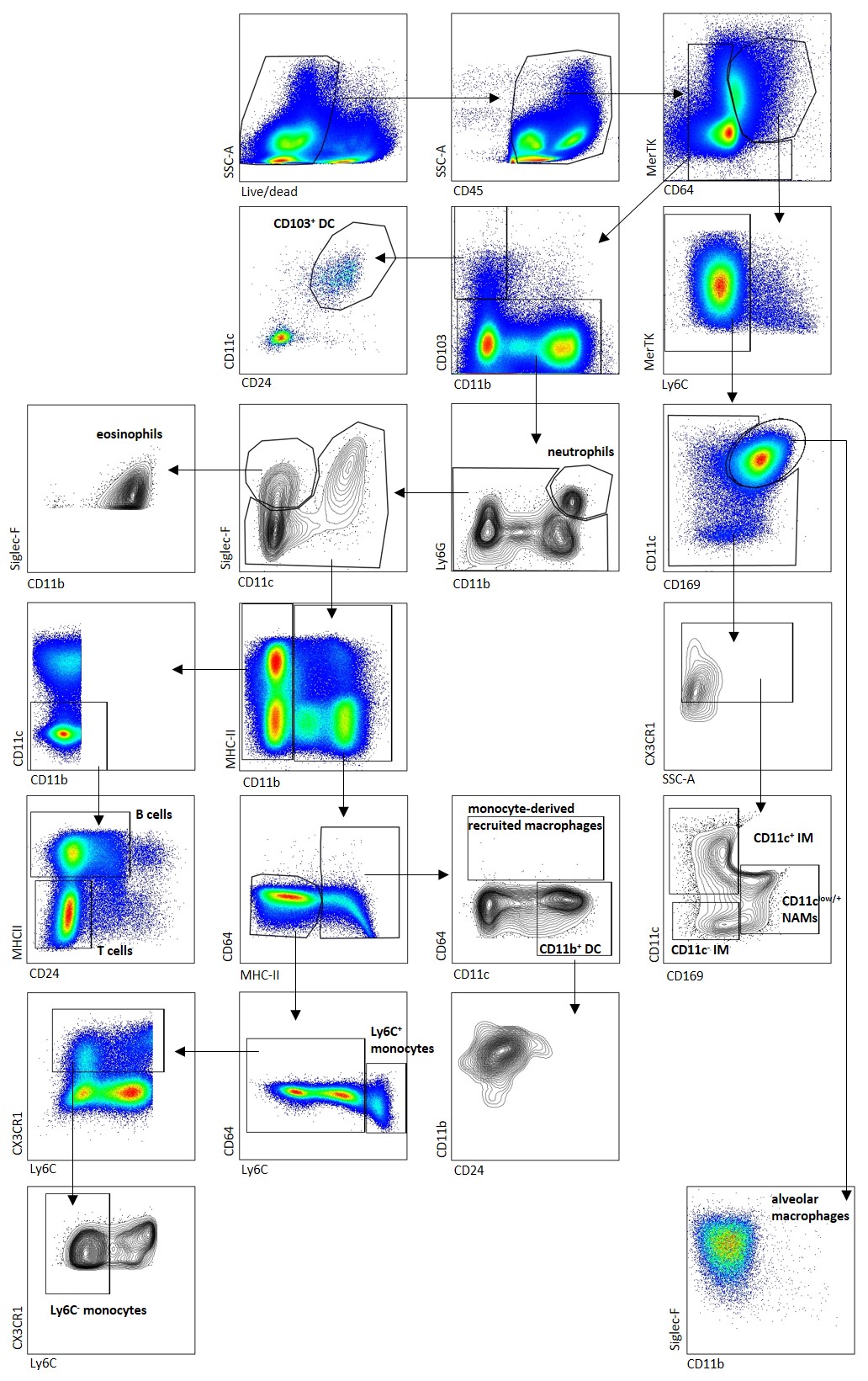
**

**Supplementary Figure 3.** Application of the gating strategy without handling of autofluorescence (AF) in the unmixing
The gating strategy shown in supplementary figure 2 was applied to spectral flow cytometry data yielded following unmixing without handling of AF. Displayed here is a concatenate of the same representative samples shown in supplementary figure 2, without AF handling.

**
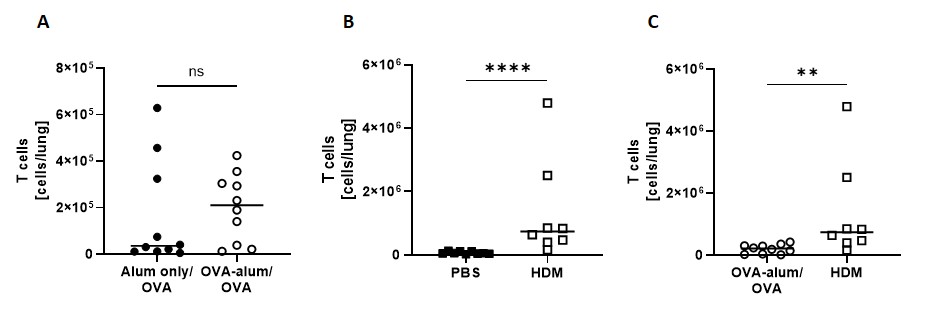
**

**Supplementary Figure 4**. OVA-AAI and HDM-AAI lead to elevated numbers of T cells in the lung. For the induction of allergic airway inflammation (AAI), mice were treated with ovalbumin (OVA) (and aluminum hydroxide (alum) for sensitization) or house dust mite extract (HDM) as described in materials and methods. Control mice were mock-treated with alum or PBS only. Lung leukocytes were analyzed for numbers of T cells (A-C). Data compiled from at least three independent experiments are shown for individual mice with the median. ***p <* 0.01*, ****p <* 0.0001*,* ns = not significant.


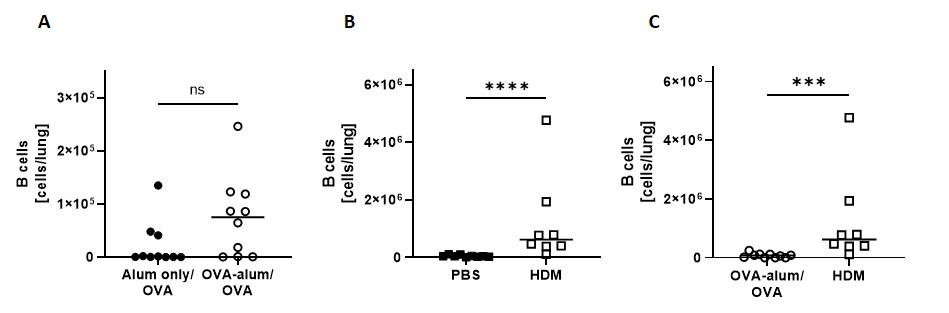


**Supplementary Figure 5**. OVA-AAI and HDM-AAI lead to elevated numbers of B cells in the lung. For the induction of allergic airway inflammation (AAI), mice were treated with ovalbumin (OVA) (and aluminum hydroxide (alum) for sensitization) or house dust mite extract (HDM) as described in materials and methods. Control mice were mock-treated with alum or PBS only. Lung leukocytes were analyzed for numbers of B cells (A-C). Data compiled from at least three independent experiments are shown for individual mice with the median. ****p <* 0.005*, ****p <* 0.0001*,* ns = not significant.

**
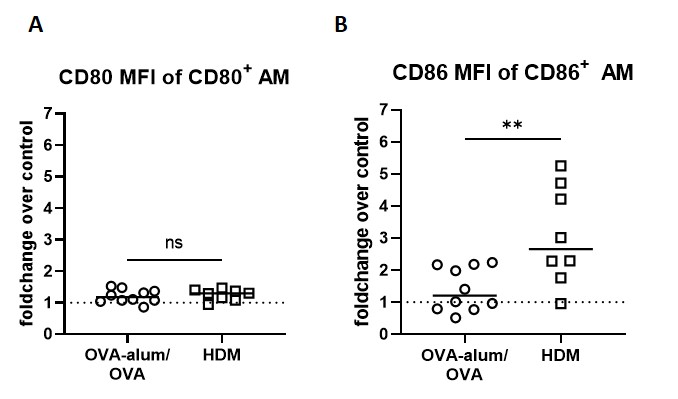
**

**Supplementary Figure 6**. Mean fluorescence intensity (MFI) of the staining for the markers CD80 and CD86 in AMs comparing OVA-AAI and HDM-AAI. For the induction of allergic airway inflammation (AAI), mice were treated with ovalbumin (OVA) (and aluminum hydroxide (alum) for sensitization) or house dust mite extract (HDM) as described in materials and methods. The MFI of alveolar macrophages (AM) for the respective marker was averaged for the control group for each separate experiment. Corresponding individual MFI-values of samples in the OVA-AAI or HDM-AAI group were compared to the median of the respective control samples, resulting in the fold change over control shown. (A) Fold change over control of the CD80 MFI of CD80^+^ AM in OVA-AAI and HDM-AAI. (B) Fold change over control of the CD86 MFI of CD86^+^ AM in OVA-AAI and HDM-AAI. Data compiled from at least three independent experiments are shown for individual AAI mice with the median. ***p <* 0.01*,* ns = not significant.
